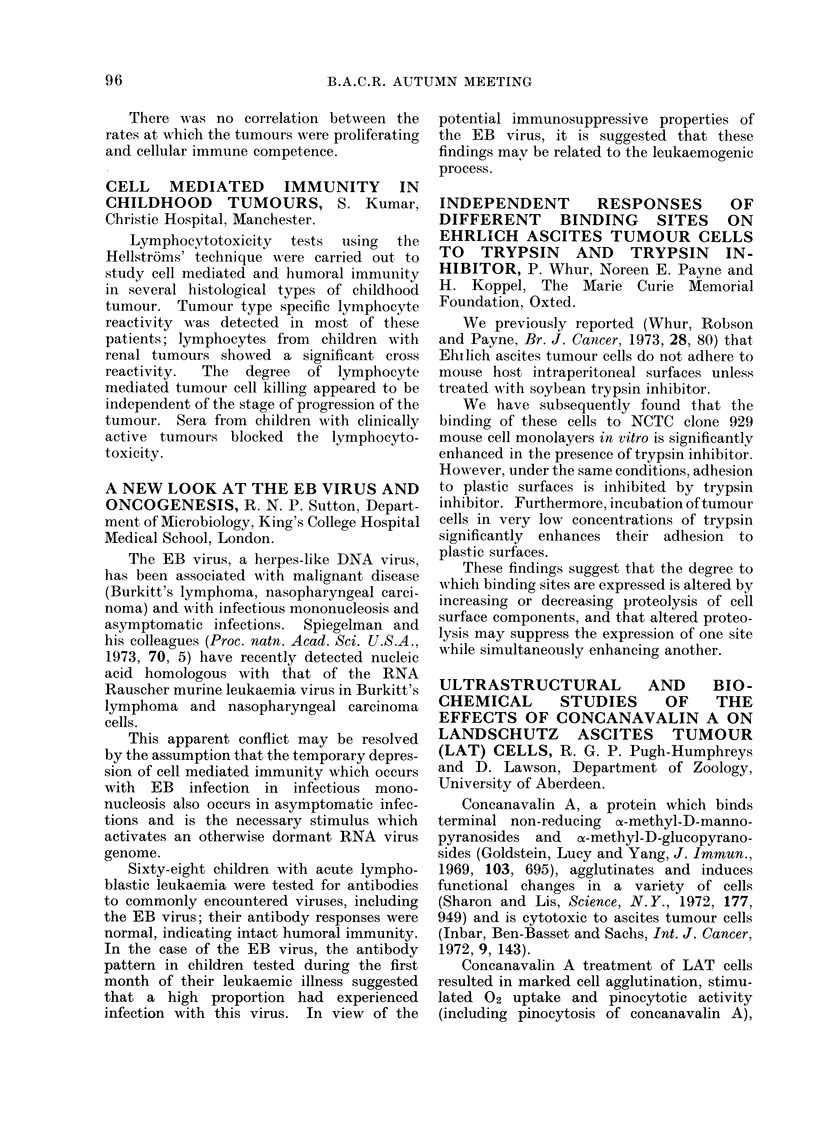# Proceedings: Independent responses of different binding sites on ehrlich ascites tumour cells to trypsin and trypsin inhibitor.

**DOI:** 10.1038/bjc.1974.28

**Published:** 1974-01

**Authors:** P. Whur, N. E. Payne, H. Koppel


**INDEPENDENT RESPONSES OF DIFFERENT BINDING SITES ON EHRLICH ASCITES TUMOUR CELLS TO TRYPSIN AND TRYPSIN INHIBITOR,** P. Whur, Noreen E. Payne and H. Koppel, The Marie Curie Memorial Foundation, Oxted.

We previously reported (Whur, Robson and Payne, *Br. J. Cancer*, 1973, **28**, 80) that Ehrlich ascites tumour cells do not adhere to mouse host intraperitoneal surfaces unless treated with soybean trypsin inhibitor.

We have subsequently found that the binding of these cells to NCTC clone 929 mouse cell monolayers *in vitro* is significantly enhanced in the presence of trypsin inhibitor. However, under the same conditions, adhesion to plastic surfaces is inhibited by trypsin inhibitor. Furthermore, incubation of tumour cells in very low concentrations of trypsin significantly enhances their adhesion to plastic surfaces.

These findings suggest that the degree to which binding sites are expressed is altered by increasing or decreasing proteolysis of cell surface components, and that altered proteolysis may suppress the expression of one site while simultaneously enhancing another.